# Appendiceal disease in hematopoietic cell transplantation

**DOI:** 10.1002/ccr3.5047

**Published:** 2022-02-03

**Authors:** Zachary Wright, Francis Essien, John Renshaw, Michael Wiggins, Alexander Brown, Michael Osswald

**Affiliations:** ^1^ Hematology/Oncology and Bone Marrow Transplant San Antonio Military Medical Center FT Sam Houston Texas USA; ^2^ Internal Medicine Travis AFB California USA

**Keywords:** appendiceal disease, chemotherapy, hematopoietic stem cell transplant, immunocompromised, medical management

## Abstract

Appendiceal diseases are rare reported complications during hematopoietic stem cell transplantation with no guidance on management in the published literature. Medical therapy may be considered in selected patients prior to surgical solutions.

## Graphical abstract

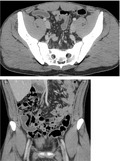

## 1 INTRODUCTION

Appendiceal diseases are rare reported complications during hematopoietic stem cell transplantation with poor management guidance. We present a case series of 5 immunocompromised patients who presented with appendiceal disease with 2 requiring surgical intervention and 3 with conservative treatment. Thus, medical management may be considered an effective treatment in poor surgical candidates.

Severe neutropenia and immunodeficiency, as either sequelae of hematologic malignancies or direct consequences of chemotherapy or hematopoietic cell transplant (HCT), increase the risk of infectious complications. Gastrointestinal infections observed in this patient population account for approximately 30% of neutropenic infections.^1^ Mortality secondary to gastrointestinal infections in neutropenic patients has only been reported in two single‐institution reviews but is consistent at 13% and 14%.^1^, ^2^ The incidence of acute appendicitis in the pediatric population with acute leukemia or lymphoma is approximately 1.5%^3^; however, the incidence is unknown for adults with hematologic malignancies or HCT patients. Acute appendicitis is, indeed, scarcely mentioned in hematopoietic cell transplant literature.^1^, ^4^, ^5^ Although uncommon, appendiceal disease is a challenging diagnostic and therapeutic problem. These patients may be afflicted with alternative diseases such as mucositis, acute GVHD of the gastrointestinal tract, neutropenic enterocolitis (typhlitis), or infectious colitis, which confounds the diagnosis. Additionally, patients may be in various stages of hematologic recovery either in the pre‐transplant or in the pre‐engraftment period and may be on additional immunosuppression for prophylaxis or treatment of graft‐vs‐host disease (GVHD). This can lead to atypical, nonlocalized pain and/or lack of peritoneal signs which may delay diagnosis. The attenuated clinical findings were apparent in one pediatric study and resulted in a 37.5% error rate in accurate diagnosis of appendicitis.^6^ These factors make it difficult to pursue invasive management given higher risk of surgical complications. Medical treatment and surgical intervention have been described in hematologic malignancies^3^, ^6^ and hematopoietic cell transplant,^2^, ^4^, ^5^ but the lack of evidence makes management challenging. We report here on five adult hematopoietic cell transplant patients treated in our institution who developed appendiceal disease at various times in their clinical course.

## 2 CASE 1

**FIGURE 1:**
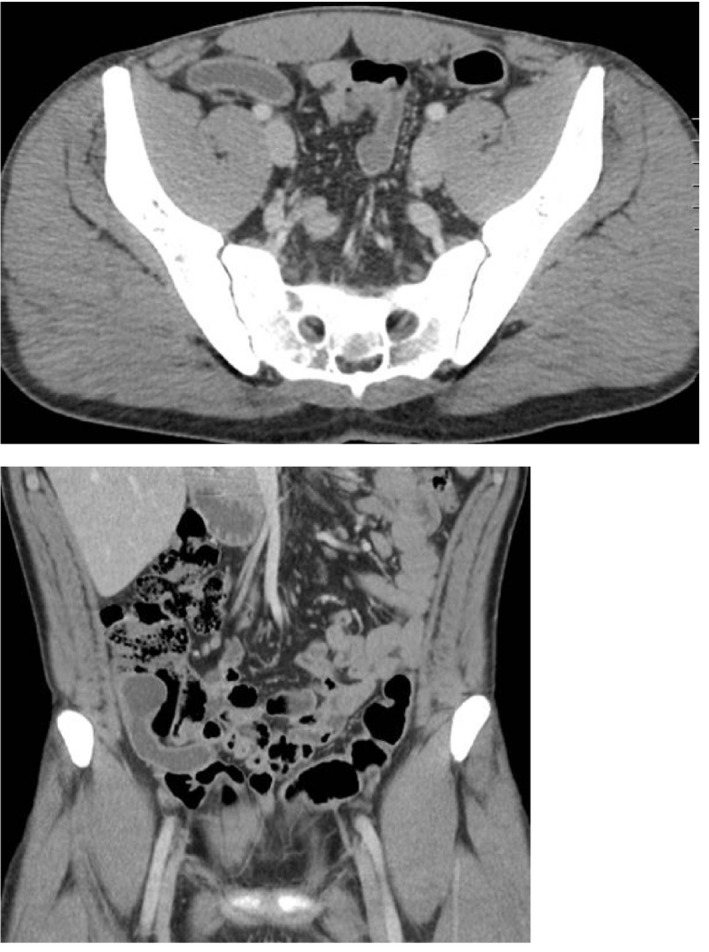
Axial (top) and coronal (bottom) views of patient 1. Findings notable for large, dilated appendix with mucosal enhancement. Pathology post laparoscopic appendectomy revealed mucinous adenoma

Patient 1 was a 23‐year‐old male with peripheral T‐cell lymphoma not otherwise specified (PTCL‐NOS) who received six cycles of cyclophosphamide, doxorubicin, vincristine, etoposide, and prednisone (CHOEP) with complete metabolic response (CR1). He was referred for high dose chemotherapy with autologous peripheral blood stem cell transplant. Restaging PET and CT showed no evidence of disease but was notable for incidental findings of dilated and enhanced appendix (Figure 1). The patient was asymptomatic with benign physical examination, and laboratory data were unremarkable. This prompted surgical evaluation which resulted in immediate preemptive laparoscopic appendectomy. Resulting pathology revealed a mucinous adenoma without high‐grade dysplasia and margins uninvolved by tumor. Transplant was postponed for 5 weeks to allow for adequate recovery. The patient went on to receive a conditioning regimen of carmustine (BCNU), etoposide, cytarabine, and melphalan (BEAM) followed by autologous stem cell transplant without any complications.

## 3 CASE 2

**FIGURE 2:**
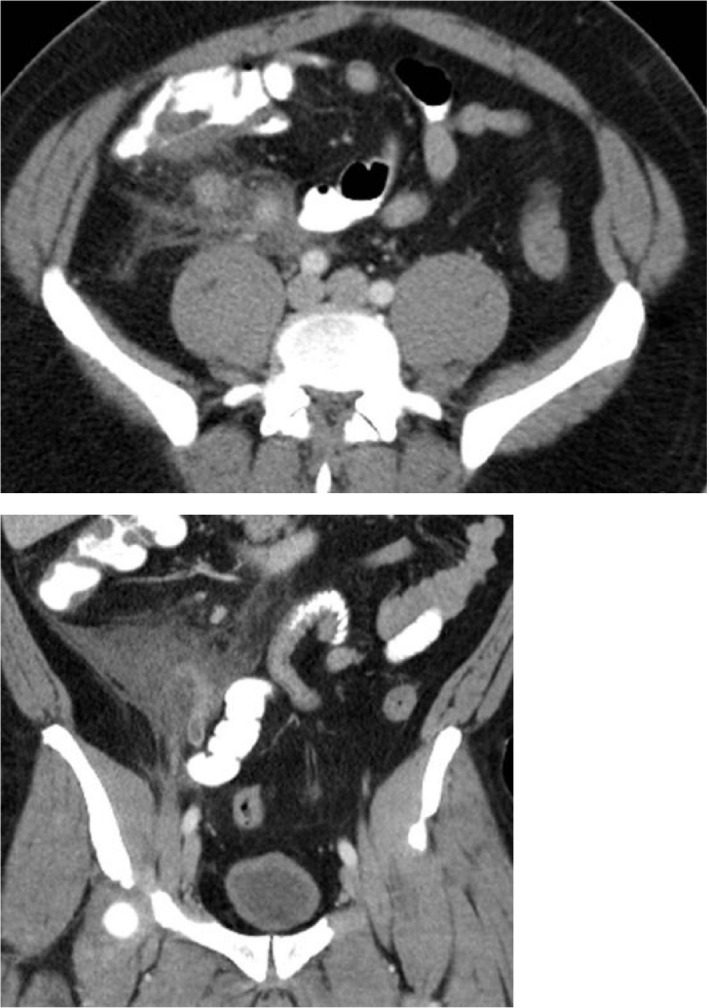
Axial (top) and coronal (bottom) views of patient 2. Findings notable for enlarged appendix with inflammatory stranding throughout the right lower quadrant sparing the cecum and terminal ileum

Patient 2 was a 49‐year‐old male with pre‐B‐cell acute lymphoblastic leukemia (ALL) who completed the first cycle of induction therapy (Hyper CVAD), which was complicated by neutropenic fever and abdominal pain. A CT revealed enlarged appendix with extensive adjacent inflammatory stranding throughout the right lower quadrant consistent with appendicitis (Figure 2). The patient was initially evaluated by general surgery who determined that he was not a surgical candidate given severe thrombocytopenia and neutropenia. He was transitioned to metronidazole and levofloxacin after initial broad‐spectrum antibiotics with piperacillin/tazobactam. He later developed perforation with abscess and lactic acidosis prompting an alternative antibiotic regimen, intravenous (IV) ertapenem. A pelvic drain was placed, and drain sample cultures revealed extended‐spectrum beta‐lactamase (ESBL) *Escherichia coli*. The patient received a prolonged course of IV ertapenem, and his cell counts recovered prior to elective laparoscopic appendectomy. He recovered, achieved CR1, and later received a myeloablative conditioning regimen of cyclophosphamide and total body irradiation (Cy TBI) for matched related donor allogeneic peripheral blood stem cell transplant. He later developed veno‐occlusive disease (VOD) and ultimately died of treatment‐related mortality on day +42.

## 4 CASE 3

**FIGURE 3:**
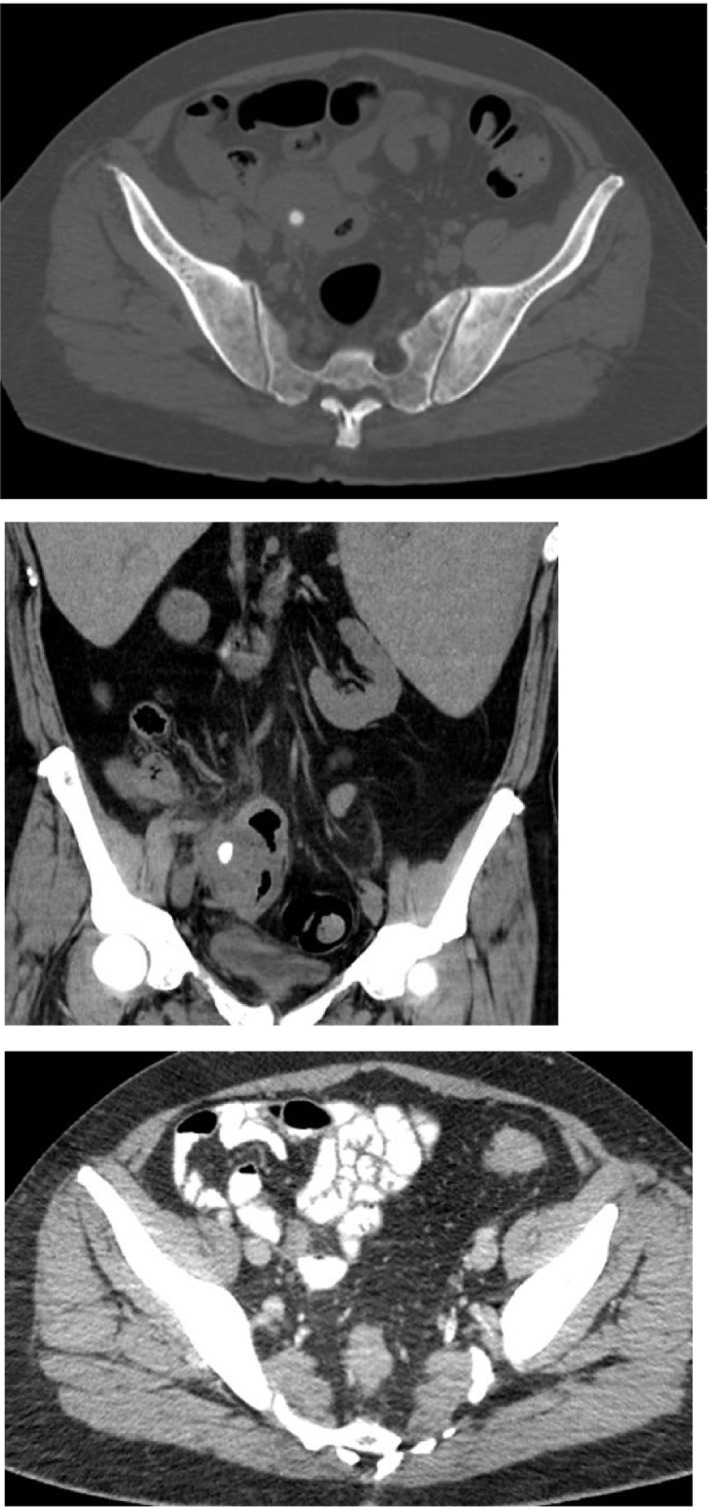
Axial (top) and coronal (middle) views of patient 3. Findings notable for enlarged appendix is with a 7 mm stone likely in the distal lumen and a 4.0 × 2.7 cm phlegmonous region intimately involved with the distal appendix. Follow‐up axial image (bottom) on day +195 show resolution of inflammation and appendicolith

Patient 3 was a 55‐year‐old male with myelofibrosis who received a reduced intensity conditioning regimen of busulfan and fludarabine with matched related donor allogeneic peripheral blood stem cell transplant. His early post‐transplant course was complicated by delayed platelet recovery and serum sickness from antithymocyte globulin GVHD prophylaxis. He presented on day +108 with right lower quadrant abdominal pain and subsequent CT showed an enlarged appendix with a 7 mm appendicolith, adjacent to phlegmon/abscess and fat stranding (Figure 3). The patient was determined not to be a surgical candidate due to thrombocytopenia and ongoing immunosuppression with tacrolimus. He received a 2‐week course of metronidazole and levofloxacin with plan to receive elective appendectomy after completion of immunosuppression or sooner if clinical status worsened. The patient clinically recovered and repeat imaging on day +195 revealed resolution of the appendicolith and inflammatory findings. Appendectomy was not pursued given lack of symptoms and resolution of radiographic findings.

## 5 CASE 4

**FIGURE 4:**
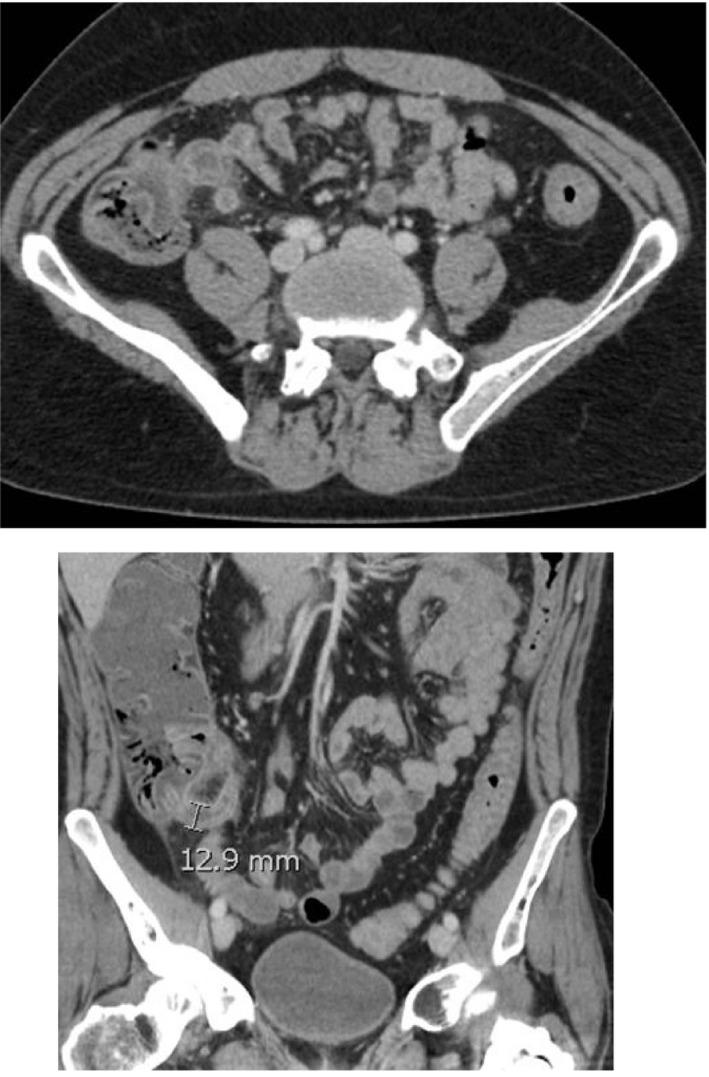
Axial (top) and coronal (bottom) views of patient 4. Findings notable for enlarged appendix with mesenteric fat stranding and terminal ileitis

Patient 4 was a 33‐year‐old male with history of diffuse large B‐cell lymphoma (DLBCL) who developed leptomeningeal relapse approximately a year after his initial therapy. He obtained a second complete remission (CR2) with a high‐dose chemotherapy regimen containing cytarabine, methotrexate, ifosfamide, and thiotepa. He received a conditioning regimen of BCNU, thiotepa, and etoposide with autologous peripheral blood stem cell transplant. He developed regimen‐related toxicity with subsequent neutropenic fever and empirically treated with cefepime. The patient noted ongoing diarrhea and right lower quadrant pain on day +9. A CT showed findings consistent with appendicitis and reactive terminal ileitis (Figure 4). A *Clostridium difficile* PCR was obtained as part of a routine institutional diarrhea evaluation and was positive. He was transitioned to piperacillin‐tazobactam and metronidazole. He was determined not to be a surgical candidate due to pancytopenia and eventually transitioned to a 2‐week course of ciprofloxacin and metronidazole. His symptoms resolved, and subsequent *C*. *difficile* PCR was negative.

## 6 CASE 5

**FIGURE 5:**
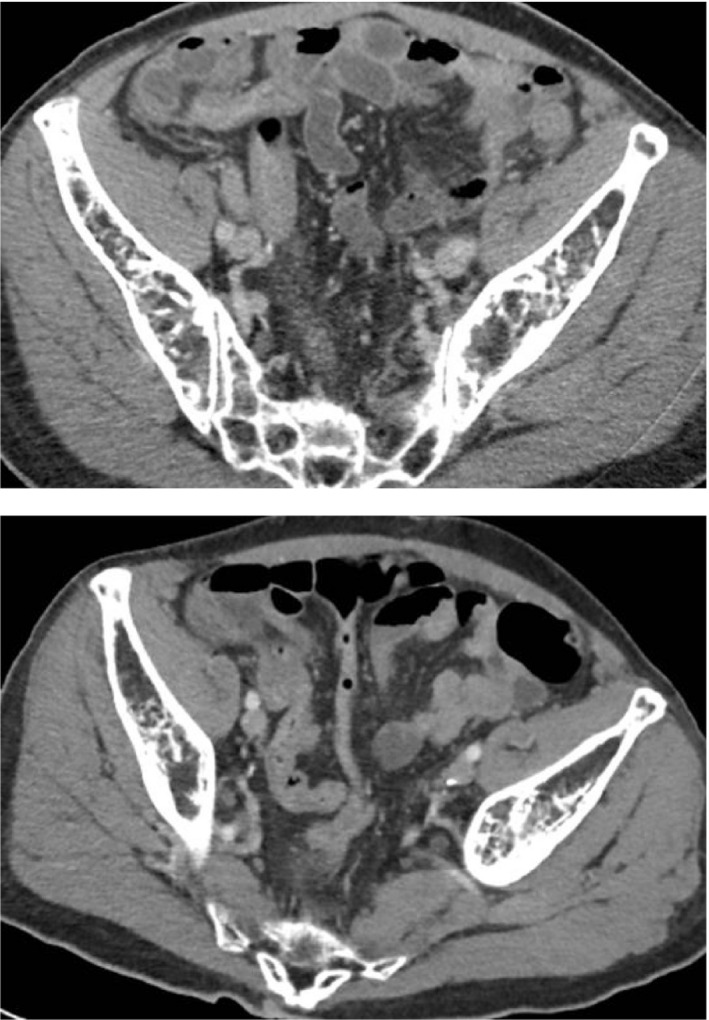
Axial (top) and coronal (bottom) views of patient 5. Findings notable for extensive small bowel and colonic mucosal hyperenhancement with wall thickening involving the descending and sigmoid colon with focal inflammation at the splenic flexure. Dilated fluid‐filled appendix with mucosal hyperenhancement

Patient 5 was a 58‐year‐old male with history of myeloma/plasma cell leukemia who obtained a partial response (PR) after 4 cycles of cyclophosphamide, bortezomib, dexamethasone (CyBorD), and later bortezomib, thalidomide, dexamethasone, cisplatin, doxorubicin, cyclophosphamide, and etoposide (VTD‐PACE). He received a conditioning regimen of standard high dose melphalan with autologous peripheral blood stem cell transplant. He developed mucositis by day +3 which peaked at grade 3 toxicity. He developed neutropenic fever on day +8 and complained of abdominal pain. A full fever workup was obtained, and patient was started on piperacillin‐tazobactam. A subsequent CT scan showed colonic mucosal hyperenhancement and wall thickening to include a dilated fluid‐filled appendix consistent with diffuse mucosal inflammation and possible appendicitis (Figure 5). Empiric antibiotic with piperacillin/tazobactam was continued for 1 week and discontinued after neutrophil engraftment. The patient recovered without complication.

## 7 DISCUSSION

All of our hematopoietic stem cell transplant patients were successfully treated for appendicitis utilizing surgical and/or medical management depending on their clinical scenario. Appendectomy, whether open or laparoscopic, remains the overall gold standard for treatment of appendicitis and in line with treatment guidelines of the American College of Surgeons and World Society of Emergency Surgery.^7^, ^8^ The use of open versus laparoscopic appendectomy in the general population is not in the scope of this review; however, there are proponents of using laparoscopic appendectomy among patients who are immunocompromised.^7^, ^9^, ^10^ Others advocate for laparoscopic appendectomy in patients with pancytopenia as it has been associated with decreased postoperative infection, hemorrhagic complications, and a lower mortality rate.^10^ One particular case series involving children with acute leukemia showed mixed use of open and laparoscopic appendectomy in patients with a mean absolute neutrophil count of 800 cells/m^3^ and boasted no intraoperative or postoperative complications.^11^ There is no data large enough to determine statistical efficacy of appendectomy among adult transplant patients, and the use of appendectomy in HCT remains anecdotal but an effective treatment for patients in various stages of hematopoietic recovery.^2^, ^4^, ^5^ Still, appendectomy in this patient population is not without its risk of infection, delayed healing, hemorrhagic complications, or operative risk based on severity of systemic disease. A multidisciplinary team should carefully consider these risks when determining whether to pursue appendectomy or conservative therapy.

Medical management without surgery is an alternative approach for the treatment of appendicitis. Medical management normally consists of bowel rest, pain management, intravenous fluids, and broad‐spectrum antibiotics with both gram‐negative and anaerobic bacteria coverage.^12^, ^13^ Multiple retrospective and randomized controlled trials have evaluated the efficacy of conservative antibiotic treatment compared to surgery in the general population. A large retrospective cohort involving 231,678 patients with appendicitis found 3236 patients who were managed nonsurgically. Only 5.9% of these patients had subsequent treatment failure which had no impact on overall mortality. After risk adjustment, mortality rates were not statistically significant between the surgical and nonsurgical patients at 0.1% and 0.3% respectively. However, hospital duration was statistically longer among the nonoperative patients (2.1 vs. 3.2 days; *p* < 0.001).^14^ A meta‐analysis involving 741 patients in four randomized controlled trials showed higher efficacy in the patients receiving surgery compared with conservative management (OR = 6.01, 95% CI = 4.27–8.46). However, surgery was associated with statistically significant higher complication rates (OR = 1.92, 95% CI = 1.30–2.85).^12^ Another meta‐analysis involving 59,448 patients in 20 retrospective studies evaluated outcomes in patients with appendiceal abscess or phlegmon who received both surgical and nonsurgical treatment. Treatment failure was noted in 7.2% of the patients who received nonsurgical therapy. Immediate surgery was associated with higher complications compared with nonsurgical treatment (OR, 3.3, CI = 1.9–5.6, *p* < 0.001).^15^ It is difficult to interpret how these results would apply to patients who additionally have neutropenia and/or immunosuppression. A case series involving five children with acute leukemia and neutropenia reported successful conservative treatment of acute appendicitis without the need for surgery. However, one patient did pursue elective appendectomy prior to bone marrow transplantation.^13^

Our experience showed that 3 of the 4 patients with neutropenia and/or on immunosuppression were successfully treated with nonsurgical management. None of these patients had recurrence or complications associated with appendicitis. The patient who failed nonsurgical therapy ultimately was found to have perforation with abscess requiring pelvic drain placement and culture revealing ESBL *E*. *coli*. The patient was maintained on ertapenem and ultimately received elective appendectomy after hematopoietic recovery and prior to transplant. The final patient received a preemptive laparoscopic appendectomy for dilated appendix which ultimately was discovered to be a benign mucinous adenoma after histologic review. As seen here, management of appendicitis in the peri‐transplant setting depends on the clinical scenario.

In pre‐transplant patients with appendicitis, elective appendectomy should be considered a means of source control prior to transplant. Although no data exist in this particular scenario, it is important to manage existing infections to reduce the risk of further infectious complications throughout the peri‐transplant period. One could argue for laparoscopic surgery in this case to decrease morbidity and potentially mitigate further delay. It is reasonable to provide a trial of nonsurgical therapy, including broad‐spectrum antibiotics, if the pre‐transplant patient is still recovering from cytopenias with prompt surgical resection upon recovery.

The pre‐engraftment patient may very well have confounding diagnoses to include mucositis, neutropenic enterocolitis, or other infectious colitis. This was the case in two of our patients: one who was suffering from mucositis and the other who was subsequently discovered to have *C*. *diff* colitis. These patients would likely benefit from a trial of nonsurgical therapy and given the potential therapeutic overlap of broad‐spectrum antibiotics and higher risk of surgical complications in the setting of pancytopenia. Reevaluation of these patients following should be considered to determine if elective appendectomy if indicated.

Post‐transplant patients may also be considered for a trial of nonsurgical therapy if they are still requiring either prophylactic or therapeutic immunosuppression for GVHD. Our post‐transplant patient had complete recovery with radiographic resolution of findings and appendicolith. However, the severity of immunosuppression varies greatly from patient to patient during this time period. There is retrospective data that notes safety among immunocompromised patients,^9^ and thus a lower threshold to pursue appendectomy in these patients is reasonable.

It is important in any of these scenarios to remain vigilant for signs of clinical deterioration. These include persistent or worsening localized abdominal pain and peritoneal signs, lack of clinical improvement with medical treatment, or hemodynamic instability/septic physiology. Further invasive therapies such as percutaneous drainage or surgical exploration may be warranted.

## 8 CONCLUSION

Appendiceal disease in hematopoietic stem cell transplant patients is rarely reported and there is little guidance in management. Our patients were all effectively treated for their appendiceal disease. Based on our experience, in pre‐transplant patients who present with appendiceal disease, there is a need to balance the risks and benefits of definitive surgical resolution of the appendiceal disease. Pre‐transplant patients with severe cytopenias from chemotherapy should be considered to receive a trial of medical therapy with plan for appendectomy after recovery but prior to transplant. Appendicitis in pre‐engraftment patients may be confounded by alternative infectious process or mucositis. In these patients, a trial of medical therapy may be considered with subsequent evaluation after engraftment. Post‐transplant patients on immunosuppression may receive a trial of medical therapy with plan for appendectomy after completion of immunosuppression.

## CONFLICT OF INTEREST

The authors report no conflict of interests.

## AUTHOR CONTRIBUTIONS

Zachary Wright M.D. and Michael Osswald M.D. involved in study conception and design. John Renshaw M.D. involved in data collection Zachary Wright M.D., Michael Wiggins M.D., and Alexander Brown M.D. involved in analysis and interpretation of results. Zachary Wright M.D. and Francis Essien D.O. drafted and prepared the manuscript. All authors discussed the results and contributed to the final manuscript.

## VERBAL CONSENT

Verbal consent was obtained from all patients for this study.

## CONSENT

Informed consent was obtained from the patient to utilize the images.

## Data Availability

Data sharing is not applicable to this article as no new data were created or analyzed in this study.
